# Successful Conservative Treatment: Multiple Atypical Fractures in Osteoporotic Patients After Bisphosphate Medication: A Unique Case Report

**DOI:** 10.1097/MD.0000000000000446

**Published:** 2015-02-06

**Authors:** Hyo-Sang Kim, Han Young Jung, Myeong-Ok Kim, Kyung-Lim Joa, Yeo Ju Kim, Su-Yeon Kwon, Chang-Hwan Kim

**Affiliations:** From the Department of Physical and Rehabilitation Medicine (H-SK, H-yJ, M-OK, K-LJ, S-YK, C-HK); and Department of Radiology (YJK), School of Medicine, Inha University, Incheon, Republic of Korea.

## Abstract

Bisphosphonates have been commonly used for the treatment of osteoporosis. However, there have been recent case reports of atypical fractures citing their long-term use, which inhibits the turnover of bone components.

A 64-year-old woman visited the outpatient clinic with pain in her right thigh and ambulation difficulty. We found fractures at both pedicles of L4 vertebra. subtrochanteric region of right femur, and left femoral shaft upon a radiologic examination. She had taken intravenous ibandronic sodium for osteoporosis over 3 years.

We changed the bishophonates to a parathyroid hormone because it was suspected that the multiple fractures were caused by the medication. Further, rehabilitation, including progressive weight bearing, was started. After 3 months of the conservative treatment, she was able to walk independently.

In conclusion, it is necessary to evaluate the possibility of atypical fractures in osteoporotic patients when they complain of lower extremity pain and to consider alternative treatments instead of bisphosphonates.

## INTRODUCTION

Bisphosphonates have been widely prescribed for the treatment of osteoporosis, and their therapeutic effects have been proved.^1,2^ Bisphosphonates are considered safe, but recent cases have shown that their long-term use may precipitate atypical femoral fractures such as subtrochanteric and diaphyseal femoral fractures.^3–5^

This case was about an osteoporotic patient who had suffered atypical fractures on both femurs and the pedicles of the lumbar spine after treatment with intravenous ibandronic sodium, 13 times, over 3 years.

Accordingly, we tried to find correlations between the atypical fractures and bisphosphonates through clinical presentations and radiologic evaluations. Unlike this case, most of the patients with atypical fractures had a medical history of long-term use of bisphosphonate for >5 years, and they usually received surgical fracture treatment.^6,7^

We report this rare case of multiple atypical fractures after bisphosphonate therapy. The patient had a successful recovery through conservative treatment.

### Case

A 64-year-old woman visited our clinic in the department of physical medicine and rehabilitation complaining of right thigh pain and both lower extremity weakness. The patient had a limping gait due to the pain in her right thigh, which had persisted for 4 months without direct traumatic episodes. It had aggravated 3 months previously due to slipping in her bathroom. Motor weakness of her right thigh was apparent. The sensation of her lower extremities was normal. Her deep tendon reflexes seemed to be hypoactive, and there was no muscular atrophy.

**FIGURE 1:**
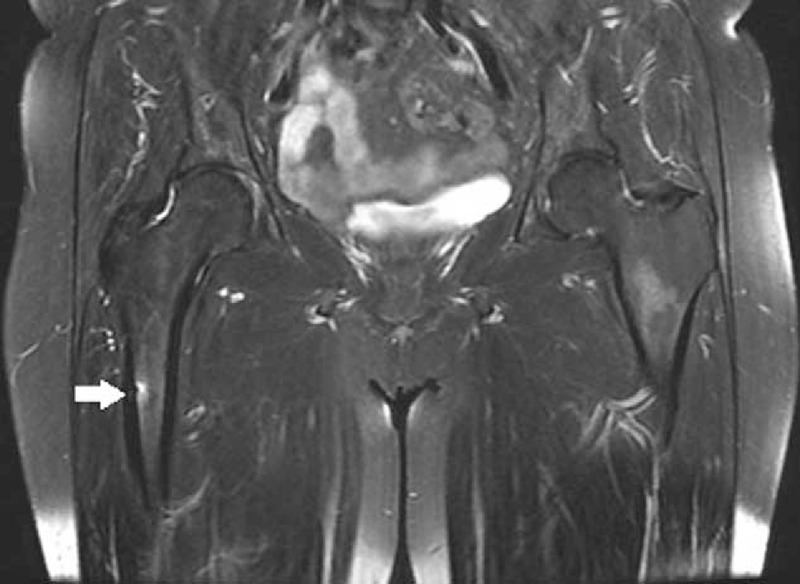
Hip MRI reveals a right femur subtrochanteric fracture (arrows). MRI = magnetic resonance imaging.

**FIGURE 2:**
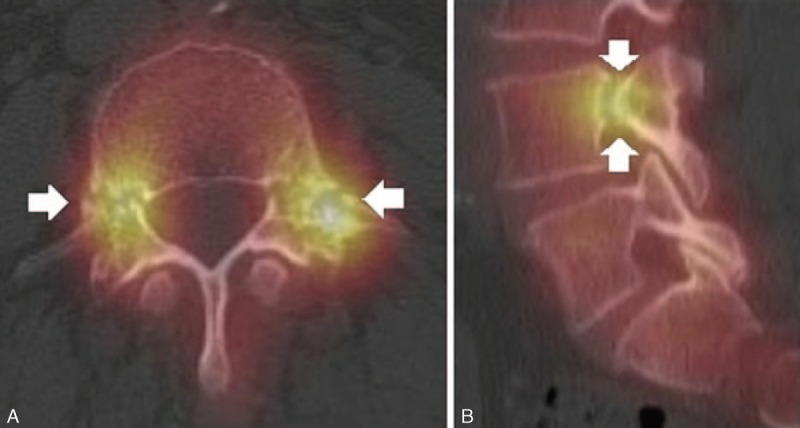
Hybrid SPECT–CT scan was conducted. (A) The SPECT-CT demonstrates the bilateral pedicle fracture through L4 (arrows). (B) The SPECT-CT parasagittal reconstruction image confirms L4 pedicle fractures (arrows). SPECT–CT = single-photon emission computed tomography–computed tomography.

**FIGURE 3:**
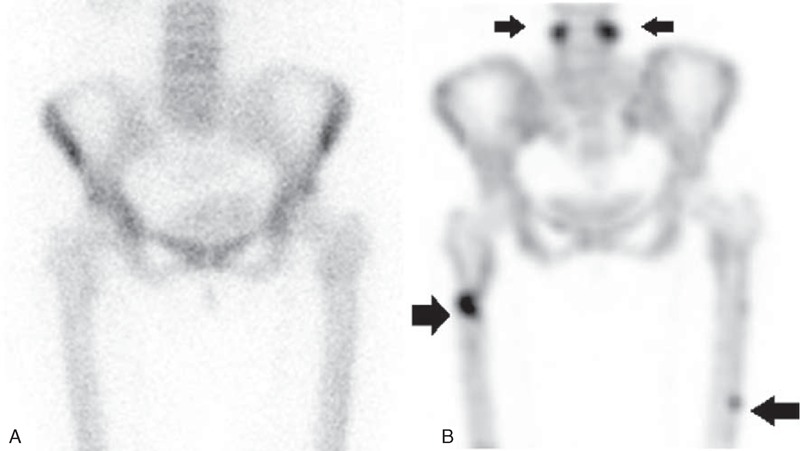
(A) Bone scan taken in 2011 shows no fractures. (B) Bone scan in 2013 shows multiple fractures including both a femur fracture and a bilateral pedicle (arrows).

We initially assumed that the weakness of her lower extremities was due to inflammatory neuropathy or lumbar herniated nucleus pulposi. However, neurophysiologic studies yielded normal results. We thus suspected a hip joint problem and a consequent magnetic resonance imaging (MRI) scan was conducted. Surprisingly, a linear fracture at the right subtrochanteric cortex and a stress fracture with bone marrow edema at the left femur shaft were observed in the hip joint MRI (Figure 1). We had not suspected fractures to be the cause of the symptoms. The patient also had a hybrid single-photon emission computed tomography (SPECT)-computed tomography (CT) scan 2 weeks later, and it revealed atypical fractures on both femurs and a bilateral fracture of the L4 pedicles as it appeared as a hot uptake (Figure 2). These fractures were found on bone scan in 2013 (Figure 3B). The patient had a hip x-ray 1 month later, and the linear fracture at the right subtrochanteric cortex was aggravated and crossed not only the cortex but also the bone marrow. We thus concluded that the pain and weakness of her lower extremities were due to atypical fractures of both femurs.

She had been diagnosed with osteoporosis 3 years before by a rheumatologist. Since then, she had received intravenous ibandronic sodium (Bonviva (Roche, Switzerland) injection), a derivative of the bisphosphonates, for osteoporosis starting in 2010, at 3 mg every 3 months, 13 times in total. Bone mineral density (BMD) by dual energy x-ray absorptiometry (DEXA) in 2010 showed the L-spine (T-score −3.0) and the femur (T-score −1.3) without fractures on a bone scan (Figure 3A). Also, she was undergoing cryotherapy and daily nisolone at 2.5 mg weekly and MTX at 5 mg for psoriatic arthritis diagnosed 2 years before for skin lesions and pain in her fingers.

A follow-up study of her BMD by a DEXA in February 2013 showed her L-spine (T-score −3.4) and her femur (T-score −1.6) at the time of the fracture diagnosis. Blood chemical tests revealed a low bone specific alkaline phosphatase of 7.5 (normal 15–41.3) μg/L and osteocalcin of 3.80 (normal 4.00–12.00) ng/mL.

**FIGURE 4:**
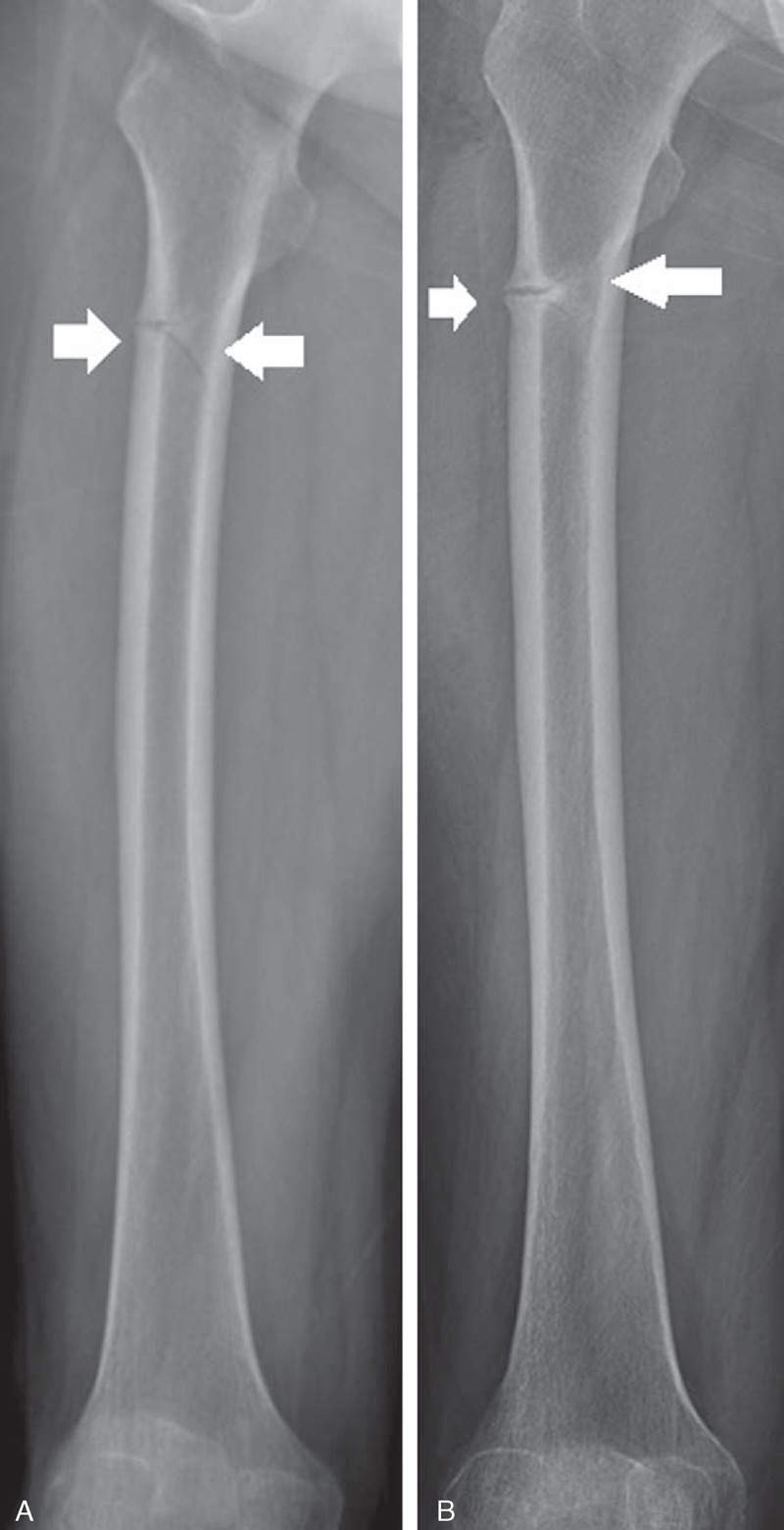
(A) Femur x-ray was conducted at the time of diagnosis (arrows). (B) Femur x-ray was conducted after conservative management (arrows).

The patient preferred conservative treatments to surgical management for the multiple fractures. We changed the medication to a monthly subcutaneous injection of a parathyroid hormone and provided her with 3-month rehabilitation programs for partial weight bearing and gait with assistive devices. The follow-up image studies showed an improved union of her femur bone fractures (Figure 4B) compared with the initial image (Figure 4A). After 3 months of the conservative treatment, she was able to walk as an improved union was observed in the x-rays without any adverse medication effects.

## DISCUSSION

Bisphosphonates inhibit bone resorption, and are widely prescribed as a treatment for osteoporosis.^1,2^ Its mechanism includes inhibiting osteoclasts and inducing their apoptosis, which eventually increases bone density as bone resorption decreases.^8,9^ Although bisphosphonates are the standardized prevention choice for osteoporosis and fractures,^10–12^ they may also reduce the bony turnover and cause atypical fractures.^3–5^

To date, the most severe known complications of bisphosphonate use is jaw necrosis, and there is a consensus of diagnosis and treatment.^13^ However, there are no treatment and diagnostic guidelines for atypical fractures despite an increase in the number of incidences.^4,14^ The incidences of atypical fractures increase as the treatment is prolonged, especially for regiments lasting >5 years.^6,7^ If an atypical fracture occurred over short-term use, as in this case, contributing factors must be investigated.

We also need to consider the correlations between the isolated fracture of the bilateral L4 pedicles and the use of bisphosphonates. The fracture at the posterior element of a vertebra may be a malignant pathologic fracture, so it is important to differentiate this from osteoporotic fractures.^15^ There have been some reports that fractures at pedicles may coincide with osteoporosis, whereas others say they do not.^16,17^ A previous case demonstrated that patients using the medication, risedronate, longer than 10 years had an isolated fracture of the bilateral L5 pedicles.^18^ Therefore, further studies may be necessary to find spinal fractures that have occurred as an atypical fracture or as an osteoporotic fracture.

We observed not only femur fractures but also pedicle fractures on bone SPECT CTs, which was different from previous case reports. Bone scans or SPECT CTs may be helpful to find incidental fractures in different body parts. It is known that a HYBRID bone SPECT CT has a high diagnostic validity in finding jaw necrosis and in differentiating other fractures, which are hard to distinguish in other simple radiologic images.^19,20^

Lastly, the application of an alternative medication, which acts on the osteoblast such as parathyroid hormone, should be considered. In this case, we changed the intravenous bisphosphonates to a parathyroid hormone, expecting that the bone turnover treatment would positively affect the bony union of the right subtrochanteric fractures. Refraining from weight bearing and the use of alternative medication resulted in a successful bony union, as observed on image studies 3 months later. Further, a large-group study is essential to validate conservative treatment for atypical fractures and establish it as a therapeutic guideline.

## CONCLUSION

Bisphosphonates are the first line of treatment for osteoporosis, and they are widely used in the world. However, there still are controversies regarding its duration of use and maintenance regiments notwithstanding their adverse effects. Physicians and patients should be aware of the symptoms, which bisphosphonates may cause such as jaw necrosis or atypical fractures. At the same time, it is important to consider not only surgery but also alternative conservative treatments for atypical fractures and establish therapeutic guidelines.
